# Risk Factors in Normal-Tension Glaucoma and High-Tension Glaucoma in relation to Polymorphisms of Endothelin-1 Gene and Endothelin-1 Receptor Type A Gene

**DOI:** 10.1155/2015/368792

**Published:** 2015-12-01

**Authors:** Dominika Wróbel-Dudzińska, Ewa Kosior-Jarecka, Urszula Łukasik, Janusz Kocki, Agnieszka Witczak, Jerzy Mosiewicz, Tomasz Żarnowski

**Affiliations:** ^1^Department of Diagnostics and Microsurgery of Glaucoma, Medical University, Chmielna 1, 20-079 Lublin, Poland; ^2^Department of Clinical Genetics, Medical University, Radziwiłłowska 11, 20-080 Lublin, Poland; ^3^Department of Internal Diseases, Medical University, Staszica 16, 20-081 Lublin, Poland

## Abstract

*The aim* of the research is to analyse the influence of polymorphisms of endothelin-1 gene and endothelin-1 receptor type A gene on the clinical condition of patients with primary open angle glaucoma.* Methods*. 285 Polish patients took part in the research (160 normal-tension glaucoma and 125 high-tension glaucoma). DNA was isolated by standard methods and genotype distributions of four polymorphisms in genes encoding endothelin-1 (K198N) and endothelin-1 receptor type A polymorphisms (C1222T, C70G, and G231A) were determined. Genotype distributions were compared between NTG and HTG groups. The clinical condition of participants was examined for association with polymorphisms.* Results*. A similar frequency of occurrence of the polymorphic varieties of the studied genes was observed in patients with NTG and HTG. There is no relation between NTG risk factors and examined polymorphisms. NTG patients with TT genotype of K198N polymorphism presented with the lowest intraocular pressure in comparison to GG + GT genotype (*p* = 0.03). In NTG patients with CC genotype of C1222T polymorphism (*p* = 0.028) and GG of C70G polymorphism (*p* = 0.03) the lowest values of mean blood pressure were observed.* Conclusions*. The studied polymorphic varieties (K198N, C1222T) do have an influence on intraocular pressure as well as arterial blood pressure in NTG patients.

## 1. Introduction

The term “glaucoma” describes a group of diseases that result in progressive and irreparable optic nerve damage characterised by typical advancing changes in the optic disc and ensuing visual field defects. Normal-tension glaucoma (NTG) is a particular kind of glaucoma with a characteristic glaucomatous cupping of the optic disc, visual field defects, open anterior chamber angle, and intraocular pressure classified as normal.

Elevated intraocular pressure is one of the most important risk factors in glaucoma development. However, its reduction in patients with normal-tension glaucoma does not entirely prevent the disease from advancing. Research done by the Collaborative Normal-Tension Glaucoma Study Group (CNTGSG) suggests other factors that play a role in the pathogenesis of glaucomatous optic neuropathy. Special attention is paid to primary vascular dysregulation since its symptoms, such as low arterial blood pressure, cold extremities, Raynaud's syndrome, and migraines, often are present in persons with normal-tension glaucoma [1].

So far, scientific research has resulted in a few theories explaining the pathomechanism of glaucomatous optic neuropathy.

Mechanical theory assumes that elevated intraocular pressure (IOP), the main risk factor in glaucomatous optic neuropathy, plays an important role in the optic nerve damage. In 1957 Von Graefe noticed that there exists a relation between elevated intraocular pressure and deformation of the lamina cribrosa [2]. Numerous studies have proven that high intraocular pressure causes cribriform plate abnormalities, which cause pressure and subsequent damage of the axons of retinal ganglion cells which pass through the plate [3].

According to the vascular theory, glaucomatous optic neuropathy is triggered by a variety of systemic and local vascular factors leading to low ophthalmic perfusion and ischemia. Among systemic factors we can list the following: hypertension and hypotension, cardiovascular diseases, diabetes, and cerebral circulation disorders [4]. Topical factors causing ischemia are as follows: low blood perfusion pressure and anatomical abnormalities of vessels supplying the eyeball [5]. Examination of the fundus of the eye reveals changes resulting from ischemia: sphincter optic disk haemorrhages, notching, focal areas of choroidal atrophy, and slower retinal flow shown by an angiogram.

Excitotoxicity theory posits that there are many processes which contribute to the development of glaucomatous optic neuropathy, for example, the toxic influence of cell mediators, neurotrophin deficiency, ischemia, or apoptosis. Ischemia, caused by autoregulation mechanism disorders and endothelium dysfunction, stimulates the release of ET-1, norepinephrine, and nitric oxide (NO), which in turn influence the blood flow in the optic disc. Subsequent reperfusion results in a considerable rise in free oxygen radicals and nitrogen compound concentration. NMDA receptor is then activated which fuels NO synthesis and radicals production. Toxic peroxynitrates, produced in the process, lead to retinal ganglion cell apoptosis.

Endothelin-1 (ET-1), discovered by Yanagisawa et al. in 1988 [6], the strongest vasoconstrictive agent in the human body, takes part in regulating ocular blood flow, the outflow of aqueous humour from the anterior chamber, and, as a result, intraocular pressure. It also assists in retinal ganglion cell apoptosis [7, 8]. Endothelin influences 2 kinds of receptors (related to protein G): ET_A_R and ET_B_R, which have the opposite effect. Numerous* in vitro* research has demonstrated that ET-1 can be found in the corneal epithelium, endothelium cells, pigmented part of ciliary body, iris, trabecular meshwork, lens, choroid, retinal pigment epithelium, retinal ganglion cells, and astrocytes [9, 10]. ET_A_ receptors have been identified in the retinal and choroidal vessels as well as in the iris, whereas ET_B_ receptors have been found in the retinal neurons, glia, and ciliary body. Both kinds of receptors can be found in the lamina cribrosa, which suggests that ET-1 plays an important role in local extracellular matrix remodelling and vascular wall stress regulation [11]. Therefore, ET-1 level and gene polymorphism might be crucial in the pathogenesis of glaucoma. The aim of this study is to find a relation between polymorphisms of ET-1 gene and ET-1 receptor type A and their influence on clinical condition of patients with normal-tension glaucoma and high-tension glaucoma (HTG).

## 2. Material and Methods

285 Polish Caucasian patients treated at the Department of Diagnostics and Microsurgery of Glaucoma in Lublin (Poland) between 2009 and 2013 took part in the study. To demonstrate the differences between the two types of primary open-angle glaucoma patients were divided into two groups: those diagnosed with normal-tension glaucoma (160 patients, including 110 women and 50 men) and those presenting with high-tension glaucoma (HTG) and intraocular pressure above 21 mmHg (125 patients, including 87 women and 38 men). Patients were accepted for the study only if they had been previously informed about the aim and scope of the research, expressed their consent for participating in the study, and met the following criteria. The investigation was conducted in compliance with the tenets of the Declaration of Helsinki.

Criteria for diagnosing high-tension glaucoma (with high intraocular pressure) are as follows: diagnosed glaucomatous optic neuropathy based on changes in the optic disc and visual field defects, open anterior chamber angle in gonioscopy, and intraocular pressure above 21 mmHg at the time of diagnosis evaluated on the basis of a 24-hour intraocular pressure monitoring. In the case of NTG intraocular pressure at the time of diagnosis was below 21 mmHg.

A comprehensive ophthalmic examination was performed in both groups. Visual acuity was tested using Snellen charts. Intraocular pressure was measured by means of an applanation tonometer. An ultrasound pachymeter was used to measure the thickness of cornea. All patients were also given a biomicroscopic examination with a slit lamp. To assess anterior chamber angle a gonioscopy was performed using a Zeiss gonioscope. Schaffer classification was applied in evaluating the width of the angle. Examination of the fundus of the eye made it possible to assess the optic disc structure, presence of peripapillary atrophy, notching of the neuroretinal rim, and optic disc haemorrhages. Moreover, patients with best corrected visual acuity equal to or better than 0.1 had their visual field analysed using Humphrey perimeter and 30-2 SITA-Fast strategy. Results were considered credible if the sum of falsely positive and falsely negative answers was lower than 15%. When assessing the results of visual field analysis, the mean deviation (MD) at the time of diagnosis (expressed in decibels, dB) was taken into account. Prior to clinical examination patients were asked whether they had been diagnosed with hypertension, hypotension, diabetes mellitus, or any cardiovascular disorders (migraine, cold extremities).

DNA was isolated from peripheral blood leukocytes by means of QIAamp DNA Blood Midi Kit (QIAGEN Inc., Germany). DNA concentration was measured using NanoDrop 2000/2000c Spectrophotometer V1.0 (Thermo Fisher Scientific). TaqMan SNP probe (Applied Biosystems) and CFX96 Real-Time PCR Detection System thermal cycler (Bio-Rad) were used to assay 4 polymorphisms of the single nucleotide of the endothelin-1 gene (K198N) and endothelin-1 receptor type A (C1222T, C70G, and G231A). DNA amplification was obtained by means of a Real-Time Polymerase Chain Reaction (details in Tables 1 and 2). Real-Time PCR Thermal Cycler is equipped with optical system which makes it possible to track PCR process in real time. To this end, fluorochrome-labelled molecular TaqMan probes (oligonucleotides of about 20–30 bp length) complementary to replicated DNA sequences and to PCR product were used. At the end of 5′ probe there is a fluorescent dye and at the end of 3′ a quencher. The dyes that were used in the study were FAM (6-carboxyfluorescein) and VIC (ABI company trademark, USA) and the quencher was TAMRA (6-carboxytetramethylrhodamine). The composition of the reaction mixture and its parameters are shown in Table 2.

K198N polymorphism of the endothelin-1 gene (rs 5370) leads to transversion of purine guanine (G) into pyrimidine thymine (T) in nucleotide 5665 in exon 5 (Lys/Asn change in codon 198). C1222T polymorphism of the endothelin-1 receptor type A gene (rs 5343), involving transition of pyrimidine cytosine (C) into thymine (T), concerns nucleotide 1363 in exon 8 in 3′ untranslated region (3′UTR). C70G polymorphism of the endothelin-1 receptor type A gene (rs 5335) is a transversion of pyrimidine cytosine (C) into purine guanine (G) and is located in 3′ untranslated region (3′UTR). This polymorphism concerns nucleotide 211 in exon 8. Point mutation changes the chemical structure of DNA; however, its function still remains unknown. Polymorphism of the EDN R_A_ G231A gene, which involves transition of purine guanine into adenine, is located in exon 1 in 5′ untranslated region (5′UTR).

In order to assess patients' arterial blood pressure rhythm a 24-hour ambulatory blood pressure monitoring was conducted using an ABPM device.

Statistical analysis of the obtained results was conducted by means of the IBM SPSS Statistica 19 and Statistica 10 software. The value of *p* < 0,05 was considered statistically significant.

## 3. Results

Differences in age and sex in both study groups were not demonstrated. More demographics data are presented in Table 3.

Table 4 shows the frequency of occurrence of particular polymorphic variations in the studied genes. The distributions in NTG and HTG groups were consistent with the Hardy-Weinberg equilibrium. Homozygous wild type GG genotype of K198N polymorphism of the endothelin-1 gene was observed in 198 patients (69.7%), homozygous mutant TT genotype in 11 patients (3.9%), and heterozygous GT genotype in 75 patients (26.4%). In male patients homozygous GG genotype was more frequent in patients with HTG rather than in patients with NTG (65.8% vs. 54%). A statistically significant difference was demonstrated in the frequency of occurrence of GG genotype in men from the study groups (*χ* ^2^ test, *p* < 0.05). GT genotype was more frequent in men with NTG rather than with HTG (40% versus 28.9%). The difference was statistically significant (*χ* ^2^ test, *p* < 0.05).

When analysing C1222T polymorphism it was observed that in both groups homozygous wild type CC genotype appeared in 100 patients (35.2%), homozygous mutant TT genotype in 41 patients (14.4%), and heterozygous CT genotype in 143 patients (50.4%). TT genotype was more frequent in men with NTG rather than with HTG (22% versus 13.2%).

There was no statistically significant difference in the frequency of occurrence of particular genotypes of C70G and G231A polymorphisms in both study groups (*χ* ^2^ test, *p* > 0.05).

When comparing both groups in terms of the frequency of occurrence of C70G and G231A polymorphisms with relation to patients' gender, no statistically significant differences were found (*χ* ^2^ test, *p* > 0.05) (Tables 5 and 6).

When comparing both groups in terms of the frequency of occurrence of particular genotypes of the researched polymorphisms, no statistically significant differences were found (*χ* ^2^ test, *p* > 0.05).

### 3.1. Influence of the Studied Polymorphisms on Clinical Condition

*(i) Intraocular Pressure*. Intraocular pressure differed statistically significantly between the two groups (Student's *t*-test, *p* < 0.05). When analysing K198N polymorphism the lowest intraocular pressure, 13.7 mmHg, was observed in the group of patients with NTG with TT genotype and the highest pressure, 17.6 mmHg, in patients with GG genotype (ANOVA test, *p* = 0.03). There were no statistically significant differences between intraocular pressure and the occurrence of particular genotypes of C1222T, C70G, and G231A polymorphisms in the study groups (ANOVA test, *p* > 0.05). Interestingly, the highest intraocular pressure was observed in patients with HTG with AA genotype of G231A polymorphism, whereas in patients with NTG and with the same genotype intraocular pressure was one of the lowest (14.8 mmHg), however, without statistical significance (Student's *t*-test, *p* > 0.05) (Table 7). 

*(ii) Visual Field*. Average value of the mean deviation (MD) at the time of diagnosis was −8.58 dB in the group of patients with normal-tension glaucoma and −13.83 dB in the group of patients with high-tension glaucoma. Average value of MD at the time of glaucoma diagnosis was statistically significant in both study groups (Student's *t*-test, *p* < 0.005). There was no relation between the stage of glaucoma and the occurrence of particular genotypes of the studied polymorphisms (Table 8).

*(iii) Influence of Gene Polymorphisms on Vascular Risk Factors (Tables 3 and 9)*. Optic disk* haemorrhages* were present in 27 patients (18.7%) from the NTG group and 4 patients (4.3%) from the HTG group (*χ* ^2^ test with Yates's correction for continuity, *p* = 0.02).

*Notching of the neuroretinal rim* was observed in 50.7% of patients (70 persons) with normal-tension glaucoma and 16.3% of patients (14 persons) with high-tension glaucoma (*χ* ^2^ test, *p* < 0.0001). Moreover, in the group of patients with NTG, notching was statistically significantly more frequent in women than in men (75% versus 25%, *χ* ^2^ test, *p* = 0.016).

*Peripapillary atrophy *was present in 21.7% of patients with normal-tension glaucoma and 13.4% of patients with high-tension glaucoma. No statistically significant changes were demonstrated in peripapillary atrophy occurrence in both study groups (*χ* ^2^ test, *p* > 0.05).

In the group of patients with NTG there was no relation between the occurrence of polymorphic variations of the studied genes and the occurrence of risk factors such as optic disc haemorrhages, notching, or peripapillary atrophy (*χ* ^2^, *p* > 0.05).

*Cold extremities* are statistically significantly more frequent in patients with NTG (75.6%) than with HTG (28.6%), (*χ* ^2^ test with Yates's correction for continuity, *p* = 0.018). What is more, in both study groups these symptoms were more common in women (women with NTG, 79.4%, women with HTG, 40.0%; *χ* ^2^ test, *p* = 0.059) rather than in men (men with NTG, 50%, men with HTG, 0%; *χ* ^2^ test, *p* = 0.19).

*Low arterial blood pressure* presented much more often in the group of patients with NTG (69.7%) than in the group of patients with HTG (16.7%). The frequency of occurrence of low blood pressure in both study groups was statistically significantly different (*χ* ^2^ test with Yates's correction for continuity, *p* = 0.039). Additionally, in the group of patients with NTG low blood pressure was much more common in women (77.8%) than in men (33.3%), *χ* ^2^ test with Yates's correction for continuity, *p* = 0.02.

In both study groups only NTG patients complained of* migraines*, and they were predominantly women (*χ* ^2^ test with Yates's correction for continuity, *p* = 0.036).

No statistically significant relation was observed between symptoms such as cold extremities, low blood pressure and migraines, and the occurrence of particular genotypes of the studied polymorphisms in both groups (*χ* ^2^ test, *p* > 0.05).

### 3.2. Analysis of the Relation between the Occurrence of Endothelin-1 Gene K198N Polymorphism and Ambulatory Blood Pressure Monitoring Results (Table 10)

Observed differences between the occurrence of GG and GT genotypes and maximum systolic pressure during the day as well as average systolic pressure during the day were close to being statistically significant. Maximum systolic pressure during the day for GG genotype was 159.4 ± 18.2 mmHg and for GT genotype 176.9 ± 42.8 mmHg, ANOVA test, *p* = 0.065. Average systolic pressure during the day for GG genotype was 127.4 ± 11.8 mmHg and for GT genotype 137.1 ± 21.6, ANOVA test, *p* = 0.064.

### 3.3. Analysis of the Relation between C1222T Polymorphism Genotypes of the Endothelin-1 Receptor Type A Gene and Ambulatory Blood Pressure Monitoring Results (Table 10)

Maximum systolic pressure during the day was observed in patients with CT genotype (176.8 ± 33.1 mmHg) and the lowest in patients with homozygous CC genotype (152.3 ± 21.2 mmHg). The difference is statistically significant, ANOVA test, *p* = 0.041. Average systolic pressure during the day was highest in patients with heterozygous CT genotype (137.4 ± 16.6 mmHg) and lowest in persons with homozygous wild type CC genotype (123 ± 14.7 mmHg). The difference was statistically significant, ANOVA test, *p* = 0.028. An interesting observation is that average diastolic pressure at night was highest in patients with homozygous mutant TT genotype (72.4 ± 15.2 mmHg) and the lowest in patients with homozygous wild type CC genotype (61.3 ± 6 mmHg). The difference was close to being statistically significant, ANOVA test, *p* = 0.055.

### 3.4. Analysis of the Relation between C70G Polymorphism Genotypes of the Endothelin-1 Receptor Type A Gene and Ambulatory Blood Pressure Monitoring Results (Table 10)

The highest average systolic pressure during the day was observed in persons with heterozygous CG genotype (135.8 ± 10.36 mmHg) and the lowest in patients with homozygous mutant GG genotype (124 ± 25.73 mmHg), ANOVA test, *p* = 0.088. The lowest average systolic pressure at night was observed in patients with homozygous GG genotype (103.8 ± 9.91 mmHg) and the highest in patients with heterozygous CG genotype (116.45 ± 15.62 mmHg), ANOVA test, *p* = 0.081.

When comparing G231A polymorphism genotypes of the endothelin-1 receptor type A gene and ambulatory blood pressure monitoring results, no statistically significant differences were shown.

## 4. Discussion

Polymorphisms of the endothelin-1 gene are in the centre of attention of many scientists from different fields of medicine. Unfortunately, among all studies available in international literature there are only few concerning their relation to the development and occurrence of glaucoma.

Many reports confirm that endothelin-1 has its part in the pathogenesis of glaucoma by influencing local blood flow in the eyeball, as well as regulating intraocular pressure and retinal ganglion cell apoptosis. Hence, it seems justified to search for a relation between ET-1 gene and ET-1 receptor type A polymorphisms and the development and occurrence of glaucomatous optic neuropathy, especially normal-tension glaucoma, whose risk factors are related to vasoconstriction.

In our research, when comparing the frequency of occurrence of particular genotypes of K198N polymorphism of the endothelin-1 gene, C1222T, C70G, and G231A of endothelin-1 gene receptor type A, no statistically significant differences were demonstrated between the study groups (normal-tension glaucoma and high-tension glaucoma), also taking into account the patients' gender. So far, not many publications referring to the above-mentioned issue have been released.

Ishikawa et al. compared the frequency of occurrence of the polymorphisms of the endothelin-1 gene and its receptors among Japanese population. 426 patients with open-angle glaucoma (including 176 persons with primary open-angle glaucoma and 250 patients with normal-tension glaucoma) and 225 healthy persons participated in the research. They were able to show statistically significant differences in the frequency of occurrence of KK genotype of K198N polymorphism in the study groups (KK genotype was more common in patients with open-angle glaucoma than in healthy patients (53.2% versus 43.8%, *p* = 0.022)) [12]. Interestingly, in our research we observed that the frequency of occurrence of homozygous wild type GG genotype (69.7%) was much higher than occurrence of mutant TT genotype (3.9%) of K198N polymorphism (107 patients with NTG and 91 with POAG) (*p* < 0.05). In addition, homozygous wild type genotype was more common in women (76.7% in POAG versus 72.7% in NTG) rather than in men (66% POAG versus 54% NTG) (*p* > 0.05). The above results might suggest a possible relation between K198N polymorphism of the endothelin-1 gene and glaucoma occurrence. Further research including healthy patients should be conducted to confirm this hypothesis.

Furthermore, Ishikawa et al. demonstrated that CC genotype of C1222T polymorphism of the endothelin-1 receptor type A gene was much more common among healthy patients (healthy patients 61.2% versus patients with glaucoma 52.6%, *p* = 0.036) [12]. In our research we observed the occurrence of homozygous CC genotype in 35.2% of patients (56 persons with NTG and 44 persons with POAG). Differences in the frequency of occurrence of the above-mentioned genotypes between our study group and Ishikawa's study group might be a result of a racial difference.

When analysing the frequency of occurrence of particular polymorphisms the researchers were not able to show their relation to gender, as was also the case in our research [12].

Genetic background of glaucoma development was the subject of a research by Kim et al., who studied polymorphisms of the endothelin-1 gene and its receptors in Korean population. Their study group were 67 patients with normal-tension glaucoma and 100 healthy persons (not treated for glaucoma). The following pattern of C1222T polymorphism genotypes was obtained: CC genotype was observed in 46.3% of patients with NTG (31 persons) and 49% from the control group (49 persons), CT genotype was present in 38.8% of patients with NTG (26 persons) and in 46% of healthy persons (46 persons), and mutant TT genotype was observed in 14.9% of patients with NTG (10 persons) and in 5% from the control group (5 persons) (*p* = 0.028). In our research we obtained a similar pattern of genotypes: CC genotype was present in 35% of patients with NTG (56 persons), CT genotype in 49.4% (79 persons), and TT genotype in 15.6% (25 persons). Later, the researchers compared the frequency of occurrence of C70G and G231A polymorphisms. They were unable to obtain statistically significant results. To sum up, on the basis of the findings the authors of the study were able to confirm a relation between C1222T polymorphism of the endothelin-1 receptor type A and the development of NTG in Korean population [14].

When comparing study groups with regard to the intraocular pressure at the time of diagnosis, a statistically significant difference was observed. In the group with normal-tension glaucoma average intraocular pressure at the time of diagnosis was 17.9 mmHg and in the group with primary open-angle glaucoma 24.65 mmHg, *p* < 0.005. The observed relation confirms an accurate choice of patients for the study groups. Similar report came from Häntzschel et al., who observed intraocular pressure of 18.8 ± 2.04 mmHg for NTG and 29.6 ± 7.9 mmHg for POAG, *p* = 0.001 [15].

Our research showed a lack of statistically significant differences between the occurrence of particular genotypes of C1222T, C70G, and G231A polymorphisms of the endothelin-1 receptor type A and the intraocular pressure at the time of diagnosis.

However, the lowest intraocular pressure at the time of diagnosis was observed in patients with normal-tension glaucoma and homozygous mutant TT genotype of K198N polymorphism (13.7 ± 2.6 mmHg) in comparison to other genotypes (GG genotype 16.8 ± 3.7 mmHg and GT genotype 16.1 ± 3.8 mmHg) (*p* = 0.03).

When analysing the relation between polymorphisms of the endothelin-1 gene and its receptors, Kim et al. noticed a statistically significant difference in the level of the intraocular pressure at the time of diagnosis of NTG between persons with AA genotype of G231A polymorphism (14.0 ± 2.8 mmHg) and GG + GA genotypes (16.2 ± 2.3 mmHg) (*p* = 0.047) [14]. We observed similar levels of intraocular pressure depending on G231A polymorphism genotypes of the endothelin-1 receptor type A gene. The lowest intraocular pressure at the time of diagnosis was in patients with normal-tension glaucoma and AA genotype (14.8 ± 4.80 mmHg) as compared to GG genotype (16.9 ± 3.4 mmHg) or GA genotype (16.0 ± 4.0 mmHg). Nevertheless, the relation was not statistically significant (*p* = 0.241). According to above data the presence of homozygous mutant AA genotype of G231A polymorphism might be a good prognostic factor for patient with NTG. The function of this polymorphism is still unknown; then further research is needed to explain it.

When comparing the occurrence of C70G polymorphism genotypes in patients with normal-tension glaucoma, Ishikawa et al. did not notice any differences in intraocular pressure between particular genotypes (GG genotype 16.5 mmHg, CC + CG genotypes 17.0 mmHg) [12]. Similar results were obtained in our research. Intraocular pressure in patients with normal-tension glaucoma presents as follows: in patients with GG genotype intraocular pressure was 16.4 mmHg, in patients with CC genotype was 17.4 mmHg, and in patients with heterozygous CG genotype was 17.6 mmHg (*p* = 0.28).

In our study glaucoma, at the time of diagnosis, was less advanced according to MD in the group of patients with normal-tension glaucoma (−8.58 ± 6.26 dB) than in patients with high-tension glaucoma (−13.83 ± 7.01 dB) (*p* < 0.0001). Häntzschel et al., while analysing visual field defects and loss of retinal nerve fibres, observed smaller visual field defect in patients with normal-tension glaucoma than in patients with primary open-angle glaucoma (NTG MD −3.69 dB versus POAG MD −9.77 dB, *p* = 0.0001) [15]. Our research seems to confirm the above observation (NTG MD −4.18 dB versus POAG MD −7.18 dB, *p* = 0.015).

The pathogenesis of optic disc haemorrhages is not yet known. It is not clear which vessels they originate from: arterioles, venules, or capillaries. One of theories claims that sudden changes of pressure in stiff scleral vessels lead to their mechanical tear. Others point to the role of primary vascular dysregulation. Elevated level of ET-1 and MMP-9 might spread from the choroid to the optic disk causing vasoconstriction, ischemia, and blood-brain barrier disruption. Such sequence of events might explain optic disk haemorrhages in patients with normal-tension glaucoma [16]. What is more, persistent vasospasms cause microinfarctions which in turn lead to visual field defects [17]. We found no statistically significant differences in the frequency of optic disc haemorrhages depending on the studied K198N polymorphisms of the endothelin-1 gene and C1222T, C70G, and G231A of the endothelin-1 receptor type A gene. Nevertheless, optic disc haemorrhages tended to occur more often in patients with wild type GG genotype of G231A polymorphism in both study groups and mutant GG genotype of C70G polymorphism in patients with high-tension glaucoma. It is worth noticing that optic disc haemorrhages do not occur in patients with mutant AA genotype of G231A polymorphism in both study groups. This might suggest that the presence of mutant AA genotype protects patients with normal-tension glaucoma against vasoconstricting factors. This is the first study about a relation between K198N polymorphisms of the endothelin-1 gene and C1222T, C70G, and G231A of the endothelin-1 receptor type A gene and the occurrence of optic disc haemorrhages.

For many years the influence of blood pressure on the development of glaucomatous optic neuropathy, especially normal-tension glaucoma, has been extensively studied. It has been pointed out in many works that hemodynamic parameters such as reduced ocular blood flow and fluctuations in ocular perfusion pressure, nocturnal fall of blood pressure, autoregulation dysfunctions, and migraines, might be the reason for ischemia and optic nerve damage [18]. Ambulatory blood pressure monitoring of patients with glaucoma makes it possible to observe the modifiable disease risk factors.

Ocular perfusion pressure, understood as a difference between average arterial blood pressure and intraocular pressure, is a crucial parameter for supplying optic nerve in necessary elements. A research conducted by Ramli et al. showed that patients with normal-tension glaucoma had much lower parameters of nocturnal blood pressure and ocular perfusion pressure than healthy persons, which only supports the great importance of ischemia theory in the pathogenesis of normal-tension glaucoma [19, 20].

On the basis of a research by Barbados Eye Study and Framingham Eye Study, diastolic perfusion pressure was acknowledged to be the most permanent vascular risk factor in the development of glaucomatous optic neuropathy [21], whereas the researchers of the Early Manifest Glaucoma Trial deemed low systolic pressure to be a factor predisposing to glaucoma progression [22].

Following Flammer, who found that nocturnal blood pressure dips are the only risk factor in patients with rapidly developing optic nerve damage in NTG, we investigate systemic blood pressure abnormalities in this group. On the basis of the interview obtained from patients and their medical history we noticed that low blood pressure was presented much more often in NTG group (69.7%) than in HTG group (16.7%) (*p* = 0.039). Taking into account the low pressure as a risk factor for normal-tension glaucoma pathogenesis, it seemed advisable to determine the relationship between systemic blood pressure values and presence of studied polymorphisms only in NTG group. We analysed the relation between the occurrence of particular genotypes of the studied K198N polymorphisms of the endothelin-1 gene, C1222T, C70G, and G231A of the endothelin-1 receptor type A, and ambulatory blood pressure monitoring results. In the case of K198N polymorphism a much higher maximum systolic blood pressure and average systolic blood pressure during the day were observed in patients with heterozygous GT genotype rather than those with homozygous wild type genotype. The difference was close to being statistically significant (*p* = 0.06). In order to confirm the preliminary observations, the study group should be larger.

In the case of C1222T polymorphism a statistically significantly higher maximum systolic blood pressure during the day and average systolic blood pressure during the day were observed in the group of patients with heterozygous CT genotype (176.8 ± 33.1 mmHg and 137.4 ± 16.6 mmHg) in comparison to homozygous CC genotype (152.3 ± 21.2 mmHg and 123 ± 14.7 mmHg) (*p* = 0.041 and *p* = 0.028). Interestingly, the highest average nocturnal diastolic pressure was observed in patients with homozygous mutant TT genotype (72.4 ± 15.2 mmHg) and the lowest with homozygous wild type CC genotype (61.3 ± 6 mmHg). The difference was close to statistical significance (*p* = 0.055).

Undoubtedly, polymorphisms of the studied genes do have an influence on the level of arterial blood pressure and its regulation. The above observations might point at the existence of autoregulation mechanisms dysfunction, vascular dysregulation, or even the dysfunction of vascular endothelium responsible for endothelin release. The mechanisms by which those polymorphisms affect function remain to be examined and clarified since functional studies are not yet available.

## 5. Conclusion

In patients with NTG and HTG a similar frequency of occurrence of polymorphic variants of the studied genes was observed. The is no relation between NTG risk factors and K198N, C1222T, C70G, and G231A polymorphisms. The studied polymorphic variants have an influence on the level of intraocular pressure and arterial blood pressure in patients with NTG. Endothelin might play an important role in glaucoma pathogenesis. Giving a better knowledge of risk factors of NTG will give us more effective tools for preventing its development or even minimizing the glaucomatous damage.

## Figures and Tables

**Table 1:** Standard amplification report.

	AmpliTaq Gold Enzyme Activation	PCR 40 cycles	
	HOLD	Denaturation	Annealing/extending
Time	10 minutes	15 seconds	1 minute
Temperature	95°C	92°C	60°C

**Table 2:** Reaction mixture composition.

Ingredient	Volume in *μ*L/well
TaqMan Universal PCR Master Mix	12.5
Stock of SNP	1.25
Isolated patient's DNA (10 ng)	11.25
Total	25

Buffer: 10 mM Tris-HCl, 1 mM EDTA, pH 8.0, DNase-free water	

**Table 3:** Demographic and clinical characteristics data.

	NTG	HTG	*p* value
Gender (females : men)	110 : 50	87 : 38	*p* > 0.05
Age (years)	72.01 ± 11,61	75.85	*p* = 0.02
BCVA	0.65	0.5	*p* < 0.05
IOP (mmHg)	17.29 ± 2.93	24.65 ± 7.78	*p* < 0.005
c/d ratio	0.79	0.8	*p* = 0.53
Haemorrhage	16%	4.3%	*p* = 0.02
Notches	50.7%	16.3%	*p* < 0.0001
PPA	21.7%	13.4%	*p* > 0.05
MD (dB)	−8.56 ± 6,26	−13.83 ± 7.01	*p* < 0.005
Cold extremities	75.6%	28.6%	*p* = 0.018
Low blood pressure	69.7%	16.7%	*p* = 0.039
Migraine	38.6%	0%	*p* = 0.036

**Table 4:** Frequency of allelic variants of polymorphisms of ET and EDN RA gene in NTG and HTG patients.

Patients (%)	K198N			C1222T			C70G			G231A		
	GG	TT	GT	CC	TT	CT	CC	GG	CG	GG	AA	GA
NTG	107 (66.9%)	7 (4.4%)	46 (28.7%)	56 (35%)	25 (15.6%)	79 (49.4%)	27 (16.9%)	44 (27.5%)	89 (55.6%)	83 (51.9%)	6 (3.8%)	71 (44.4%)
HTG	91 (73.4%)	4 (3.2%)	29 (23.3%)	44 (35.5%)	16 (12.9%)	64 (51.6%)	21 (16.9%)	36 (29%)	67 (54%)	65 (52.4%)	5 (4%)	54 (43.5%)

**Table 5:** Frequency of allelic variants of polymorphisms of ET and EDN RA gene in female and male NTG patients.

NTG	K198N			C1222T			C70G			G231A		
Patients (%)	GG	TT	GT	CC	TT	CT	CC	GG	CG	GG	AA	GA
F	80 (72.7%)	4 (3.6%)	26 (23.6%)	40 (36.4%)	14 (12.7%)	56 (50.9%)	18 (16.4%)	32 (29.1%)	60 (54.5%)	55 (50%)	4 (3.6%)	51 (46.4%)
M	27 (54%)	3 (6%)	20 (40%)	16 (32%)	11 (22%)	23 (46%)	9 (18%)	12 (24%)	29 (58%)	28 (56%)	2 (4%)	20 (40%)

**Table 6:** Frequency of allelic variants of polymorphisms of ET and EDN RA gene in female and male HTG patients.

HTG	K198N			C1222T			C70G			G231A		
Patients (%)	GG	TT	GT	CC	TT	CT	CC	GG	CG	GG	AA	GA
F	66 (76.6%)	2 (2.3%)	18 (20.9%)	31 (36%)	11 (12.8%)	44 (51.2%)	12 (14%)	27 (31.4%)	47 (54.7%)	3 (50%)	4 (4.7%)	39 (45.3%)
M	25 (65.8%)	2 (5.3%)	11 (28.9%)	13 (34.2%)	5 (13.2%)	20 (52.6%)	9 (23.7%)	9 (23.7%)	20 (52.6%)	22 (57.9%)	1 (2.6%)	15 (39.5%)

**Table 7:** Maximum intraocular pressure (IOP) according to the allelic variants of studied polymorphisms.

IOP (mmHg)	K198N			C1222T			C70G			G231A		
	GG	TT	GT	CC	TT	CT	CC	GG	CG	GG	AA	GA
NTG	17.6	13.7	17	16.6	16.4	16.4	17.4	16.4	17.6	16.9	14.8	16
HTG	19.5	24.5	25.8	26.6	21.5	25.5	27.7	21.9	25.6	25.1	33	22.7

**Table 8:** Mean deviation at the moment of diagnosis (dB).

	NTG		HTG		Student's *t*-test	
	M	SD	M	SD	*t*	*p*
Mean deviation (dB)	−8.58	6.26	−13.83	7.01	5.708	0.000

**Table 9:** Relation between glaucoma risk factors and polymorphic variants in NTG patients.

NTG												
Patients (%)	K198N			C1222T			C70G			G231A		
	GG	TT	GT	CC	TT	CT	CC	GG	CG	GG	AA	GA
Haemorrhages	21.2% (21)	16.7% (1)	15% (6)	18% (9)	9.1% (2)	22.3% (16)	15.4% (4)	20.5% (8)	30% (15)	23% (17)	0% (0)	15.6% (10)
Notches	20% (1)	49.5% (48)	64.1% (25)	49% (24)	54.5% (12)	54.2% (38)	48% (12)	51.3% (19)	54.4% (43)	52.1% (38)	50% (3)	53.3% (33)
Low blood pressure	59.1% (13)	100% (1)	90% (9)	58.3% (7)	60% (3)	81.3% (13)	57.4% (4)	58.3% (7)	85.7% (12)	68.8% (11)	0% (0)	75% (12)
Cold extremities	67.9% (19)	100% (1)	83.3% (10)	62.5% (10)	71.4% (5)	83.3% (15)	66.7% (6)	62.5% (10)	87.5% (14)	71.4% (15)	0% (0)	78.9% (15)
Migraine	32.6% (14)	100% (1)	53.8% (7)	33.3% (7)	44.4% (4)	40.7% (11)	50% (6)	29.4% (5)	39.3% (11)	32.3% (10)	50% (1)	45.8% (11)

**Table 10:** Relation between mean blood pressure (BP) (mmHg) and polymorphic variants in NTG patients.

NTG	K198N			C1222T			C70G			G231A		
	GG	TT	GT	CC	TT	CT	CC	GG	CG	GG	AA	GA
Maximum systolic BP day	159.4	—	176.9	152.3	157.5	176.8	157	155.5	173.3	160.6	167	172.2
Mean systolic BP day	127.4	—	137.1	123	126.3	137.4	125.85	124	135.8	128.14	136.16	113.46
Mean diastolic BP day	75.3	—	80.2	76.83	77.85	77.02	76.55	75.65	78.14	76.54	80.5	77.5
Mean systolic BP night	111.98	—	112.96	105.4	115.05	115.05	111.7	103.8	116.45	113.6	114.83	109.7
Mean diastolic BP night	65.3	—	67.07	61.3	72.4	65.4	69.2	60.5	66.89	66.2	65.16	65.6
